# From Tool to Social Actor: A Systematic Review of the Psychological Mechanisms Through Which Conversational AI Reshapes the Employee Experience

**DOI:** 10.3390/bs16091704

**Published:** 2026-09-21

**Authors:** Li Liu, Ruijuan Zhang, Xin Su

**Affiliations:** 1 School of Management, China Women’s University, Beijing 100101, China; liuli@cwu.edu.cn (L.L.); zhangrj@cwu.edu.cn (R.Z.); 2 School of Economics and Management, Beijing University of Posts and Telecommunications, Beijing 100876, China

**Keywords:** conversational AI, generative AI, employee experience, anthropomorphism, systematic review, human–AI collaboration

## Abstract

The rapid workplace diffusion of conversational artificial intelligence (CAI) introduces a new social actor, yet existing reviews often neglect the intrapsychic processes through which employees construe these systems. This systematic review synthesizes the emerging psychological mechanisms suggested by the literature through which CAI reshapes the employee experience. Following PRISMA guidelines, we searched the Web of Science database (up to February 2026) and analyzed 84 SSCI-indexed studies based on the sociotechnical systems theory, the Computers as Social Actors paradigm, and the Job Demands–Resources model; the corpus quality was appraised using the Mixed Methods Appraisal Tool. Findings reveal that while instrumental framing remains dominant, a meaningful theoretical turn has emerged among a minority of studies, where conversational artificial intelligence is increasingly conceptualized as a partner or supervisor fundamentally driven by anthropomorphic cues. The synthesis identifies a double-edged reshaping of work: cognitively, human intelligence augmentation competes with skill threat; emotionally, constant support contrasts with social fabric erosion; and career-wise, inclusion coexists with work alienation and generative AI loafing. These dimensions are psychologically coupled, with patterns indicating that cognitive threats co-occur with emotional anxiety and moral expediency. While limited by cross-sectional primary evidence, the review provides an integrated conceptual model illustrating proposed conceptual pathways that connect multi-layered antecedents to employee outcomes, which could be adopted to guide future longitudinal validation. By shifting the focus from productivity to psychological experience, this study offers theoretical foundations for human–AI collaboration and outlines a future research agenda on trust dynamics and algorithmic fairness.

## 1. Introduction

### 1.1. A New Social Actor in the Workplace

A profound and evolving psychological transition is unfolding in contemporary organizations as they adapt to the workplace integration of conversational AI. When an employee opens ChatGPT, Microsoft 365 Copilot, or an internal chatbot to draft a report, answer customer questions, or support a subordinate, the interaction is not experienced as a conventional software command. It is experienced as a conversation with turn-taking, coherence, and at times apparent empathy (Baygi & Huysman, 2026; Nguyen, 2026). As Microsoft (2024) reports in its Work Trend Index, 75% of global knowledge workers now use generative artificial intelligence (AI) in daily tasks, yet nearly half adopted it within the preceding six months, representing a diffusion speed that has outpaced both organizational policy and psychological theory.

This conversational interface, which we refer to throughout as conversational artificial intelligence (CAI), represents more than a technological substitution. CAI systems manifest increasing levels of social presence (Short et al., 1976) and anthropomorphic cues, such as names, first-person pronouns, empathic phrasing, and adaptive learning that reliably activate employees’ interpersonal schemas (Epley et al., 2007; Nass & Moon, 2000; Pillai et al., 2024). The resulting psychological transition is both subtle and consequential: employees no longer simply use AI; they collaborate with, rely on, distrust, and in some cases emotionally attach to it (Gkinko & Elbanna, 2022; Le et al., 2025). Understanding this shift is therefore a question about the psychology of work in a post-generative AI era.

Three features of the current moment distinguish it from previous waves of workplace automation. First, conversational artificial intelligence systems are encountered directly by end users rather than through specialist intermediaries. In contrast, earlier information technologies, including enterprise resource planning systems, decision-support software, and predictive-analytics platforms, were mediated by specialists, encountered through tightly scripted interfaces, and understood by employees as back-office infrastructure. Second, they are accessed through open natural-language interaction rather than fixed menus and scripted transactions. Third, employees confront them as counterparts in conversation rather than as back-office infrastructure. Sarkar (2026) argues that this shift changes the psychological locus of work from using a system to working with a system. The implication is not only technical. It affects trust, accountability, and the meaning of competent work. What is at stake is not the adoption of another productivity tool but a quiet renegotiation of what it means to be a competent, connected, and ethically accountable employee.

The psychological gravity of this moment is amplified by the structure of contemporary knowledge work. CAI has entered knowledge work at an unprecedented speed. Within roughly two years of ChatGPT’s public release, it has been integrated into the routines of recruitment, performance feedback, knowledge search, creative composition, and emotional counseling (Budhwar et al., 2023; Hughes et al., 2026). At the same time, employees are often left to improvise the boundaries of appropriate delegation, trust, and disclosure. Because CAI produces fluent human-register outputs, employees may also over-extend trust, reduce verification, or redirect help-seeking away from colleagues and toward the system (Baygi & Huysman, 2026; Nguyen, 2026). These features jointly turn workplace CAI into a field experiment in the psychology of human–machine sociality, an area that requires theoretical articulation beyond the adoption-intention paradigm that still dominates much of the field (Pillai et al., 2024).

### 1.2. Theoretical Perspectives

We draw on three complementary theoretical strands. First, Sociotechnical Systems (STS) theory (Trist & Bamforth, 1951; Cherns, 1976) treats organizations as jointly optimized socio-technical arrangements. When a new technology enters work, it changes not only task execution but also patterns of trust, help-seeking, and mutual recognition. From this perspective, CAI raises a direct question about the social fabric of work, which Baygi and Huysman (2026) operationalized as the density of interpersonal help relations vulnerable to being replaced by human–AI exchanges.

Second, the Computers Are Social Actors (CASA) paradigm (Nass & Moon, 2000; Reeves & Nass, 1996) and the anthropomorphism framework (Epley et al., 2007) explain how a conversational interface becomes a social counterpart in the employee’s mind. Even when users know they are interacting with software, linguistic fluency, apparent affect, and persona continuity can activate social scripts and elicit trust, reciprocity, attachment, moral expectation and disappointment. Nguyen (2026) provides direct support for this logic by showing that anthropomorphic features in a company generative AI system increase employee engagement and knowledge contribution through perceived social presence.

Third, the Job Demands–Resources (JD-R) model (Bakker & Demerouti, 2017; Demerouti et al., 2001) helps explain why CAI produces both enabling and harmful consequences. As a job resource, CAI can offload cognitive load and build creative problem finding (Lin et al., 2024). As a job demand, it can generate prompt burden, skill threat anxiety, and work alienation (Hai et al., 2025).

Three additional psychological considerations deepen these perspectives. Conservation of Resources theory (Hobfoll, 1989) complements the JD-R model by predicting that employees facing AI-induced skill threat enter a resource-protection mode. In this state, they defend their remaining expertise through vigilant investment or avoidance, a mechanism consistent with the GenAI loafing pattern observed by Saluja et al. (2025). Self-Determination Theory (Ryan & Deci, 2000) posits that the long-term motivational effects of CAI are contingent upon the perception of the technology as either fostering or obstructing autonomy. This aligns with findings of Zhang et al. (2025) that generative AI use simultaneously promotes both incremental and radical creativity through distinct intrinsic-motivation pathways. Finally, Social Identity Theory (Tajfel & Turner, 1986) enhances the CASA perspectives by suggesting that when CAI is integrated into the ingroup of “colleagues,” the distinction between human and non-human coworker becomes psychologically fluid. This integration has implications for loyalty, reciprocity, and perceived group continuity that remain empirically underexplored (Le et al., 2025).

Integrating these perspectives yields the following proposition: Through anthropomorphic cues (CASA), CAI becomes embedded in the socio-technical fabric of organizations (STS); subsequently, via the dual pathways of resource–gain and demand–cost (JD-R), it simultaneously enables and erodes employees’ cognitive, emotional, and ethical experiences. This integrative proposition structures our research questions and analytical architecture, which are empirically articulated through the synthesis of 84 studies below.

### 1.3. Research Gaps and Research Questions

While the intersection of AI and human resource management (HRM) has obtained significant academic attention, existing systematic literature reviews reveal critical theoretical and empirical gaps. Table 1 contrasts five published SLRs with the present review and shows a recurring pattern where prior syntheses map AI applications, strategic themes, ethical risks, or sustainability domains, but they do not systematically center the lived psychological experience of employees interacting with conversational AI as a social actor, nor do they map the underlying mechanisms shaping this transition.

Three theoretical gaps in the extant literature motivate this review. First, published reviews have mapped AI across HR functions, strategic adoption, and organizational levels, but they largely retain an AI-as-infrastructure assumption. Vrontis et al. (2022) organize the field around advanced technologies, AI, and robotics in HRM strategies and activities; Jatobá et al. (2023) cluster the literature into strategic HR, recruitment, training, and future of work; and Úbeda-García et al. (2025) identify six fundamental strategic research themes through bibliometric mapping, ranging from automation and predictive analysis to the personalization of the employee experience. Even when employee well-being, trust, or technostress appear in these reviews, they are nested within broad field-level themes rather than analyzed as a role transition in which employees begin to treat conversational AI (CAI) as a digital colleague or social actor (Le et al., 2025). No prior synthesis has systematically characterized the psychological profile of this shift or its anthropomorphic antecedents (addressed by RQ1).

Second, existing reviews treat ethical, social, and experiential strands in a segmented way. While Filippelli et al. (2026) proposed a three-dimensional well-being schema (emotional, social, cognitive), the ethical dimension remains under-theorized as a constitutive part of employee experience. This fragmentation is also visible across the published SLRs. Kekez et al. (2025) provide a focused review of bias and discrimination in AI-enabled HRM, but their contribution is intentionally bounded to conceptual definitions, topic distributions, and positive-versus-negative implications of fairness-related harms. Alherimi et al. (2025), by contrast, review AI in Green HRM and map technologies, sectors, and performance effects, yet employee experience appears mainly through sustainability and adoption lenses. Bankins et al. (2024), in their multilevel review of AI in organizations, likewise called for a micro-psychological agenda but provided no operationalized coordinate system. A framework that integrates cognitive, emotional–social, and career–ethical dimensions, and articulates their cross-dimensional coupling, is still missing (addressed by RQ2).

Third, published reviews are rich in thematic mapping but stop short of a process model linking antecedents, psychological mechanisms, and consequences. Jatobá et al. (2023) differentiate positive and negative approaches to AI adoption; Úbeda-García et al. (2025) identify strategic themes and recurring challenges such as technostress, trust, and fairness; and Alherimi et al. (2025) catalog implementation factors such as organizational readiness, leadership support, and ethical governance. Yet these drivers and consequences remain cataloged rather than theorized as an employee-level antecedent–process–outcome chain. Likewise, the opinion pieces of Budhwar et al. (2023) and Dwivedi et al. (2023) foregrounded multiple promises and perils of generative AI but did not construct a testable antecedent–process–outcome model. The field therefore lacks a synthesis specifying which individual- and organizational-level antecedents push employees onto which psychological path, and under what conditions the double-edged effects of human–AI interaction tilt toward flourishing or harm (addressed by RQ3).

Accordingly, we come up with three interlocking research questions, structured in a progressive logical sequence:

RQ1 (role evolution; CASA lens): How has CAI evolved from an automation tool to a partner or supervisor in employees’ perceptions, and what anthropomorphic mechanisms underlie this transition?

RQ2 (multi-dimensional experience; Integrative STS and JD-R perspectives): How is the employee experience reshaped across cognitive, emotional–social, and career–ethical dimensions through human–AI collaboration?

RQ3 (antecedents and double-edged outcomes; Integrative STS and JD-R perspectives): Which individual- and organizational-level antecedents drive this reshaping, and what positive (enabling) and negative (dark-side) consequences follow for employees’ behavior and the social structure of work?

The three questions are progressive. RQ1 characterizes the new actor (what); RQ2 maps its operating structure on employees (how); RQ3 traces the antecedent–outcome chain (why and with what consequences).

### 1.4. Contributions and Roadmap

A clarification of terminology regarding artificial intelligence, generative artificial intelligence, large language models, and conversational artificial intelligence is warranted before proceeding. These concepts represent distinct hierarchical and functional domains within the literature. Artificial intelligence serves as the overarching field. Generative artificial intelligence represents a specific subfield dedicated to systems capable of creating new content. Large language models function as the underlying deep-learning algorithmic architecture that powers advanced text-based generation. In contrast, conversational artificial intelligence refers primarily to the interface layer. It encompasses systems designed specifically for natural language dialog. While early conversational systems relied on basic rule-based programming, modern conversational agents are predominantly powered by large language models. The present review focuses specifically on conversational artificial intelligence (Hughes et al., 2026; Pillai et al., 2024). This focus is chosen because the interactive conversational interface is the specific technological affordance that activates the psychological mechanisms motivating our review. Anthropomorphic attribution and interpersonal script recruitment rely on conversational turn-taking and linguistic fluency regardless of the underlying algorithmic architecture. To ensure conceptual precision regarding borderline technologies such as artificial intelligence appraisal systems or recruitment algorithms, we established a strict boundary. These systems were only included in our corpus if they operated through a direct natural language conversational interface such as an artificial intelligence interview chatbot. Purely algorithmic back-office systems lacking a conversational interface were systematically excluded because they do not trigger the interpersonal schemas central to this review.

This review advances the AI-at-work literature through three interconnected theoretical contributions. Fundamentally, this review integrates the existing macro-level technology adoption literature with micro-level psychological perspectives. It extends current understanding by documenting how CAI becomes a social counterpart in employees’ mental models, detailing both the resulting psychological costs and benefits. To capture this experiential complexity, the review introduces a three-dimensional coordinate system encompassing cognitive, emotional–social, and career–ethical dimensions. This framework not only operationalizes the call of Bankins et al. (2024) for micro-level research but also extends Filippelli et al.’s (2026) well-being schema by elevating the ethical dimension to a peer construct. Beyond this structural mapping, our synthesis articulates a set of emergent dark-side phenomena that were largely unforeseen by prior opinion-paper agendas. These unintended consequences include GenAI loafing (Saluja et al., 2025), workplace cheating (Song et al., 2025), expediency (Hai et al., 2025), and social fabric rupture (Baygi & Huysman, 2026). Following this introduction, Section 2 details the methodology and Section 3 describes the resulting literature corpus. Section 4 presents the synthesized themes, leading into a comprehensive discussion in Section 5. Finally, Section 6 concludes the review by proposing a forward-looking research agenda.

## 2. Methods

### 2.1. Search Strategy

This systematic review adheres to the PRISMA 2020 guidelines (Page et al., 2021). Following the methodology of prior systematic reviews in this domain (e.g., Úbeda-García et al., 2025; Liang et al., 2026), we limited our search exclusively to the Web of Science (WoS) Core Collection (SSCI/SCI-E/ESCI) database. Consequently, this manuscript represents a systematic review of a restricted Web of Science corpus. This initial decision was made to maintain a strict focus on the peer-reviewed literature within organizational and psychological disciplines, though it inherently bounds the scope of our synthesis. The search was conducted on 9 February 2026. The protocol was not pre-registered, a limitation we revisit in Section 5.4. The Boolean search string combined two concept sets:

TS = ((“chatbot*” OR “Chat bot*” OR “conversational Agent” OR “Virtual Assistant*” OR “Digital Assistant*” OR “conversational AI” OR “generative AI” OR “ChatGPT” OR “large language model*” OR “LLM”) AND (“human resource” OR “HRM” OR “Personnel Management” OR “workforce” OR “employee*” OR “Talent Management” OR “Staff*”))

The initial search yielded 1210 records.

### 2.2. Inclusion Criteria and Screening

**Figure 1:**
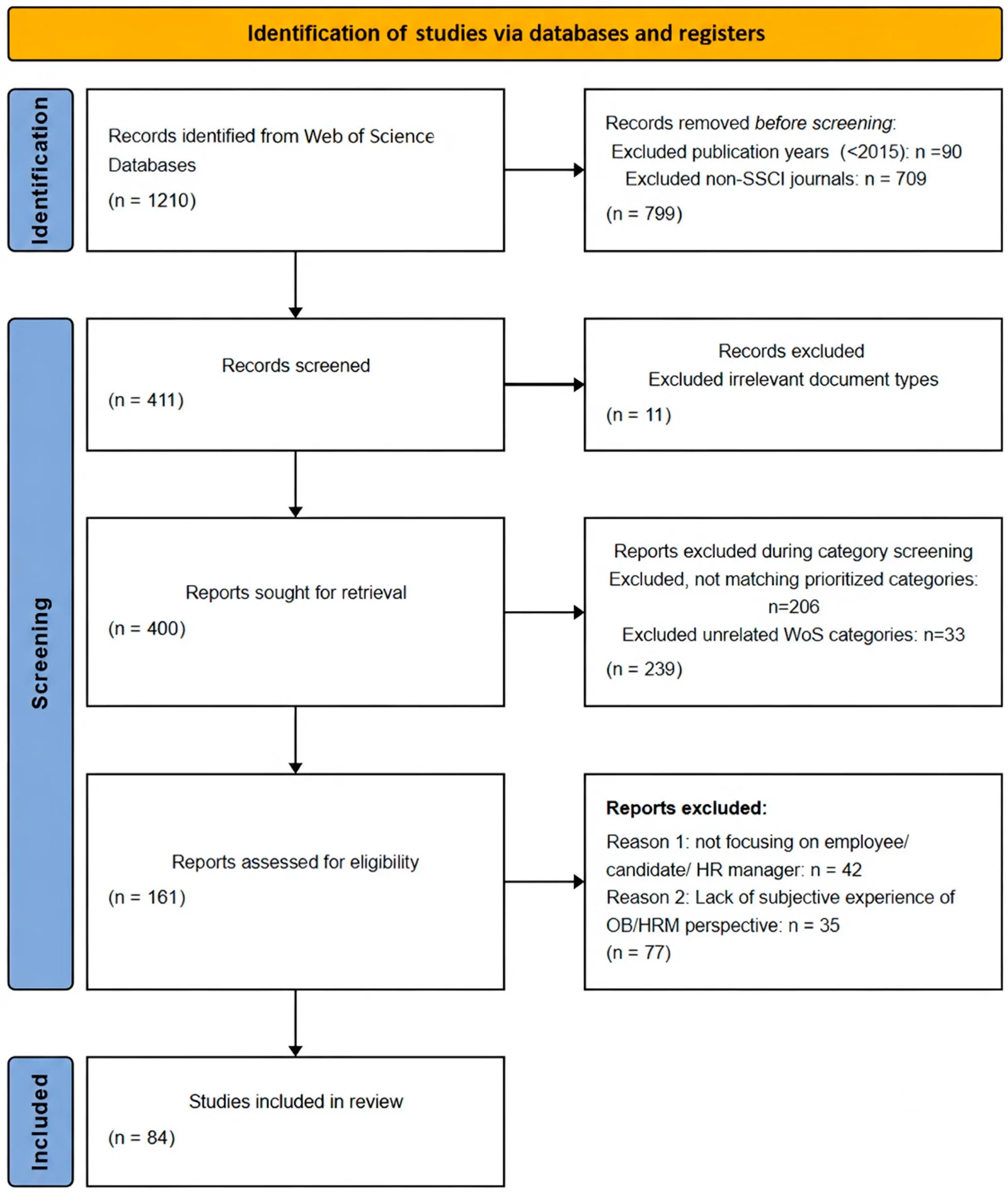
PRISMA 2020 flow diagram for the systematic review.

During the bounded database screening phase, we sequentially applied several structural filters, as shown in Figure 1. We initially set the publication window to 2015–2026 (*n* = 1120) to capture early conceptualizations of digital agents and strictly retained records indexed in the SSCI (*n* = 411). However, during the screening process, no studies published between 2015 and 2019 met the strict eligibility criteria regarding the micro-psychological employee experience of conversational AI. Consequently, the actual timeframe of the final reviewed corpus spans 2020 to 2026. The corpus was further restricted to English-language peer-reviewed articles, review articles, and early-access papers (*n* = 400).

Next, to ensure thematic relevance, we conducted a disciplinary refinement using Web of Science subject categories. We retained records from domains closely aligned with organizational behavior and technology adoption, specifically Management, Business, Applied Psychology, Information Science & Library Science, Computer Science Information Systems, and Industrial Relations & Labor (*n* = 194). Conversely, we explicitly excluded structurally misaligned fields such as clinical medicine, industrial/civil engineering, urban planning, and telecommunications, thereby reducing the pool to 161 records.

Finally in the manual eligibility assessment phase, full-text versions of all 161 records were successfully retrieved and thoroughly assessed. Two reviewers independently conducted a comprehensive title, abstract, and full-text review. Any disagreements were resolved through a consensus discussion. Borderline cases were adjudicated by strictly verifying the presence of a natural language conversation interface. This manual evaluation excluded an additional 77 out-of-scope papers, resulting in a final high-quality corpus of 84 studies. As detailed in the PRISMA flow diagram, 42 reports were excluded for not focusing on the direct human party such as employees or human resource managers, and 35 were excluded for lacking a subjective psychological experience perspective.

### 2.3. Theory-Driven Coding Framework

Two researchers (the authors) developed a 16-dimension coding framework grouped into five analytic categories, deductively anchored in the three theoretical lenses (STS/CASA/JD-R) and inductively refined through a ten-paper pilot, as shown in Table 2. The framework reflects three methodological commitments. First, the unit of analysis was fixed as the direct human party of the human–AI interaction (employees, job applicants, or HR managers) to avoid conflating HR professionals’ experience with employees. Second, we distinguished employee-end dark-side behaviors (GenAI loafing, alienation, cheating, social fabric rupture) from technology-end boundary conditions (AI hallucination, knowledge-based incompleteness). Third, the framework deliberately includes a career–ethical dimension alongside cognitive and emotional–social dimensions identified by Filippelli et al. (2026), because roughly one-third of studies in our corpus substantively engaged with algorithmic fairness, alienation, or workplace cheating.

Coding was performed independently by two researchers following a rigorous protocol to ensure reliability. A detailed coding dictionary defining all 16 dimensions, including specific criteria for varying levels of anthropomorphism and distinct employee experience categories, is provided in Supplementary Material S1. When studies included more than one artificial intelligence role or outcome, we coded the primary construct representing the core theoretical contribution or the highest level of anthropomorphic agency. This approach was chosen because capturing the peak level of social cueing is necessary to test the Computers as Social Actors hypothesis. In many multi-role designs, lower-agency systems are utilized strictly as baseline control conditions, while the highly anthropomorphic agent represents the focal experimental treatment. Prioritizing the primary, high-agency construct can capture the core theoretical mechanism while maintaining a consistent unit of analysis and preventing the duplicative coding of a single study. The initial intercoder agreement reached 93.6 percent, yielding a Cohen’s kappa of 0.88, which indicates strong reliability. Minor discrepancies were subsequently resolved through a structured consensus mechanism.

To strengthen interpretability, we also recorded the theoretical orientation, sampled population, and relationship to the three EX dimensions (cognitive, emotional and social, and career and ethical) for each study. Approximately half of the empirical studies explicitly invoked formal psychological, organizational, or information systems theories. The most frequently adopted frameworks included technology acceptance models (such as TAM and UTAUT), Affordance Theory, the Job Demands–Resources (JD-R) model, and Social Exchange Theory. Another substantial portion of the literature implicitly mobilized these constructs without formal theoretical anchoring. Furthermore, nearly a quarter of the empirical studies were atheoretical in a strict sense, reporting primarily descriptive, practical, or design-oriented findings.

For the present review, we treated all three strata as legitimate contributions but synthesized them at different levels of theoretical abstraction. Specifically, explicitly theorized studies directly informed the integrated conceptual model. Implicitly theorized studies supplied corroborative evidence for the structural patterns, whereas atheoretical studies furnished ecological grounding and contextual richness. This graded synthesis strategy aims to honor the full range of evidence while avoiding the methodological overreach of extracting causal theoretical conclusions from purely descriptive data.

### 2.4. Quality Appraisal and Corpus Rigor

Given the highly interdisciplinary and rapidly evolving nature of this topic, the Mixed Methods Appraisal Tool was adapted to focus on methodological transparency and source credibility. Two reviewers independently performed the study-level quality appraisal and resolved any discrepancies through discussion. Quantitative studies were evaluated on sampling strategy and measurement validity. Qualitative studies were assessed on methodological clarity and data richness. Conceptual papers were evaluated on logical argumentation and theoretical contribution.

While all 84 studies met the baseline inclusion threshold for methodological clarity, they exhibited varying degrees of evidentiary weight which we accounted for during the synthesis phase. Specifically, studies relying on self-reported cross-sectional designs were given lower evidentiary weight for causal inference. In contrast, higher evidentiary weight was assigned to findings derived from surveys, longitudinal designs, or randomized field experiments, which directly informed our causal propositions.

To ensure structural transparency, we explicitly categorized the 84 reviewed papers into distinct document types: (1) empirical studies (*n* = 59 out of 84, 70.2%), which include strict quantitative (*n* = 31 out of 84, 36.9%) or mixed method designs, such as PLS-SEM, field experiments, etc. (*n* = 16 out of 84, 19%) and in-depth qualitative case studies (*n* = 12, 14.3%); (2) conceptual papers (*n* = 15 out of 84, 17.9%), which proposed novel theoretical frameworks without empirical data; and (3) review-style contributions (*n* = 8 out of 84, 9.5%), which offered secondary syntheses of broader AI integration in workplaces.

Furthermore, the credibility of the findings is bolstered by their publication in top-tier outlets across Human Resource Management, Information Systems, and Organizational Behavior. By explicitly tracking research designs in our coding matrix, we ensured that the synthesis remained sensitive to the varying evidentiary weight of quantitative, qualitative, and conceptual contributions.

## 3. Descriptive Results

To enhance transparency and support future research reproducibility, a comprehensive table detailing all 84 included studies is provided in Supplementary Material S2. This file documents the study characteristics, methodological designs, and key psychological findings for each retained article.

### 3.1. Publication Trajectory and Methodological Mix

**Figure 2:**
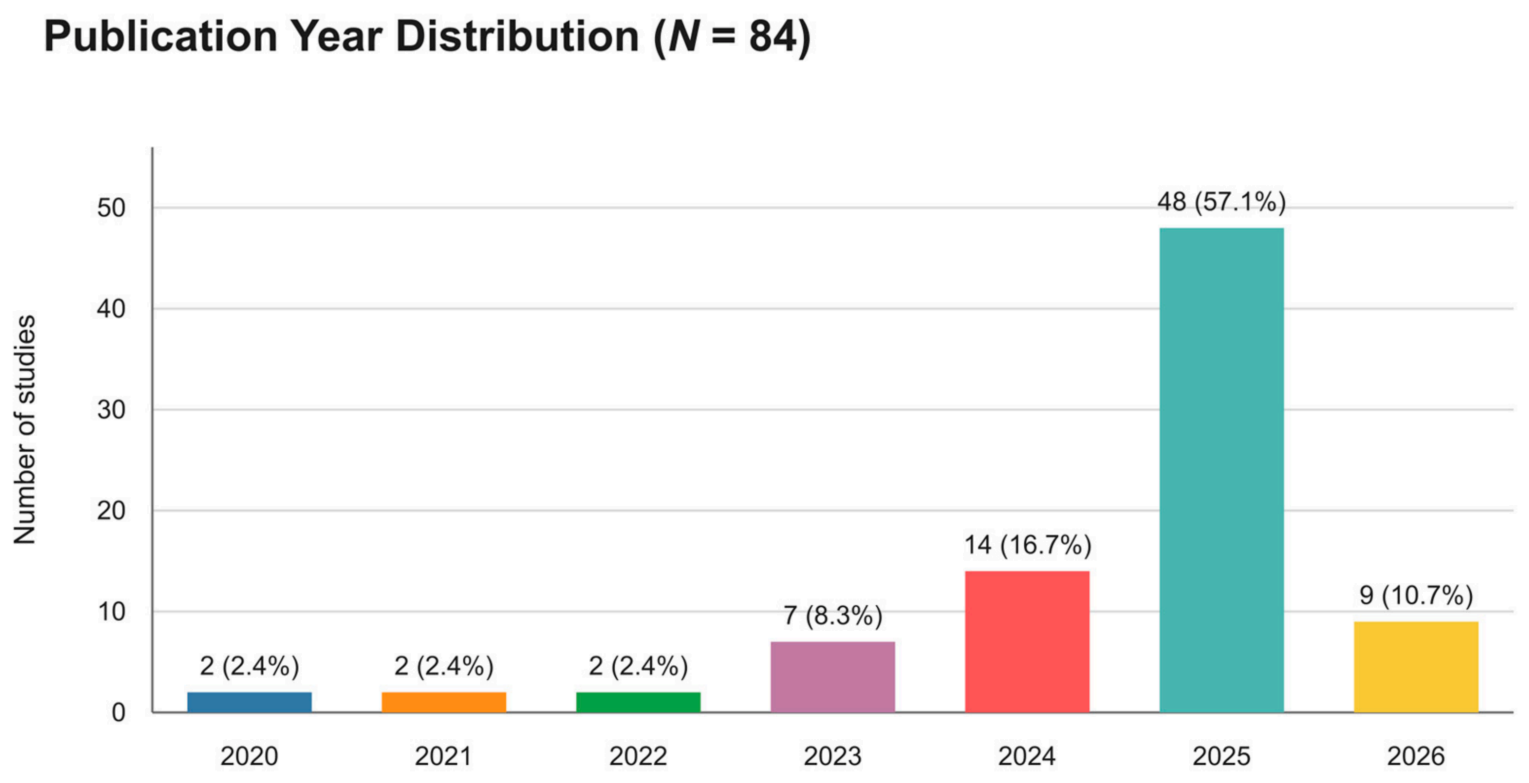
Publication trend of research on CAI and employee experience (2020–2026).

The 84 studies display a sharply accelerating publication trajectory, as illustrated in Figure 2. The corpus includes two studies each in 2020, 2021, and 2022. This baseline rises to seven publications in 2023, 14 in 2024, and 48 in 2025, with a further nine early-access publications already indexed for 2026. Combined, the period from 2024 to 2026 accounts for 84.5% of the entire corpus. This pattern closely synchronized with the public release of ChatGPT in late 2022 and the subsequent wave of scholarly response, which was initially exemplified by rapid-turnaround opinion pieces such as Budhwar et al. (2023).

**Figure 3:**
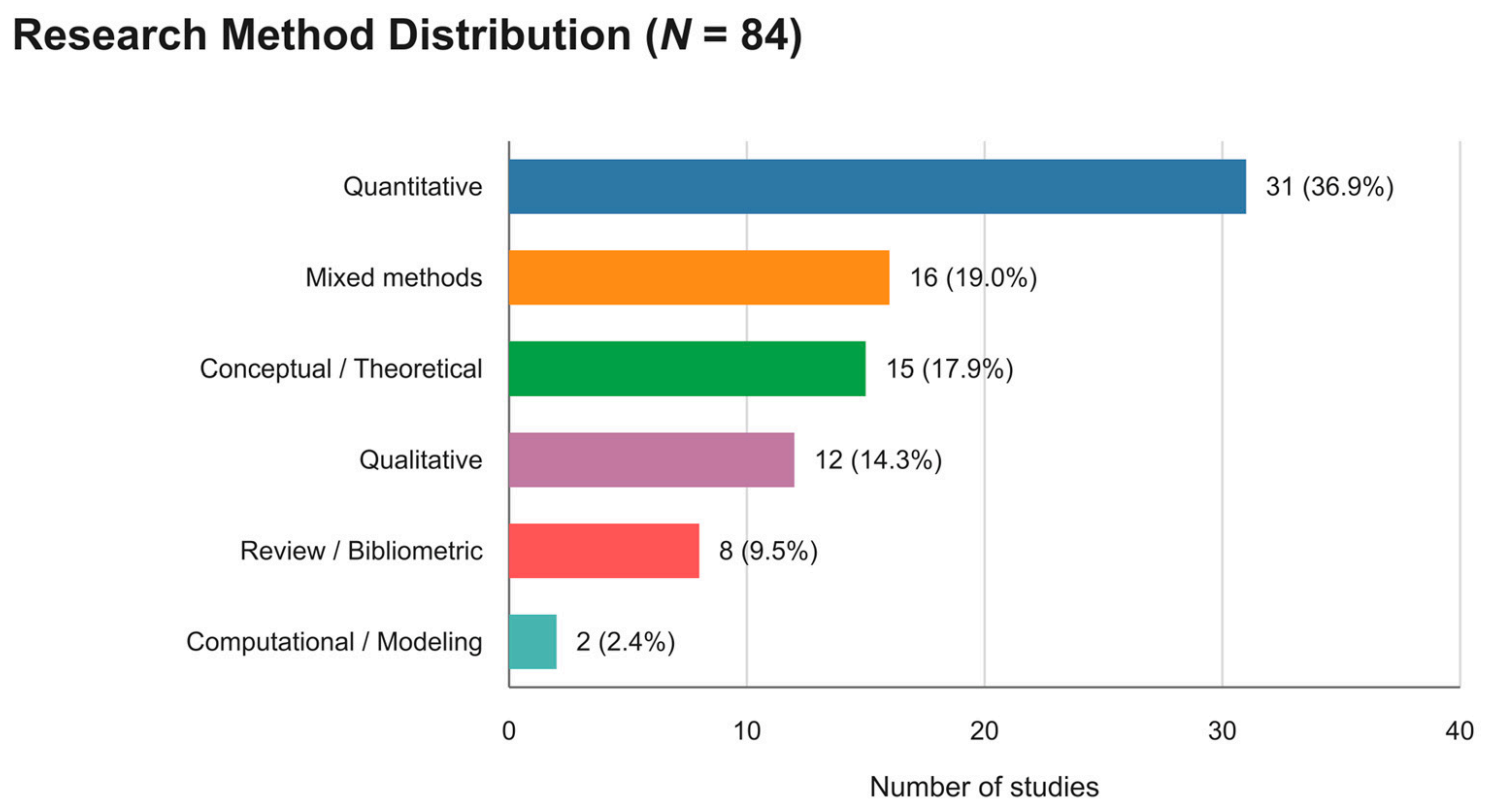
Distribution of research methods across included studies (*N* = 84).

Regarding the methodological mix shown in Figure 3, empirical designs constitute most of the reviewed literature. This strong empirical density indicates that the field has rapidly moved beyond early exploratory agenda-setting and into the testing of concrete propositions, thereby providing a defensible evidence base for the thematic synthesis below. However, this volume does not imply uniform causal strength. Given the inclusion of cross-sectional designs, conceptual papers, and reviews, the strength of direct causal inference remains tentative across the current literature.

### 3.2. Cross-Disciplinary Outlets and the Role Shift

The corpus spans approximately 50 SSCI-indexed outlets displaying a long-tailed distribution. The *International Journal of Information Management* leads with seven articles, followed by *Organizational Dynamics* with five. Several prominent journals published three articles each, including the *Journal of Innovation & Knowledge*, *International Journal of Selection and Assessment*, *Human Resource Management*, *The International Journal of Human Resource Management*, *Personnel Review*, *Journal of Organizational Change Management*, and *European Journal of Innovation Management*. Three distinct intellectual clusters emerge from this distribution including Information Systems journals, organizational behavior and general management journals, and human resource management and applied psychology journals. Pure psychology outlets remain a small minority. A core motivation for the present review is to translate cross-disciplinary evidence into a psychologically coherent framework that occupational health and industrial-organizational psychologists can productively adopt and extend.

**Figure 4:**
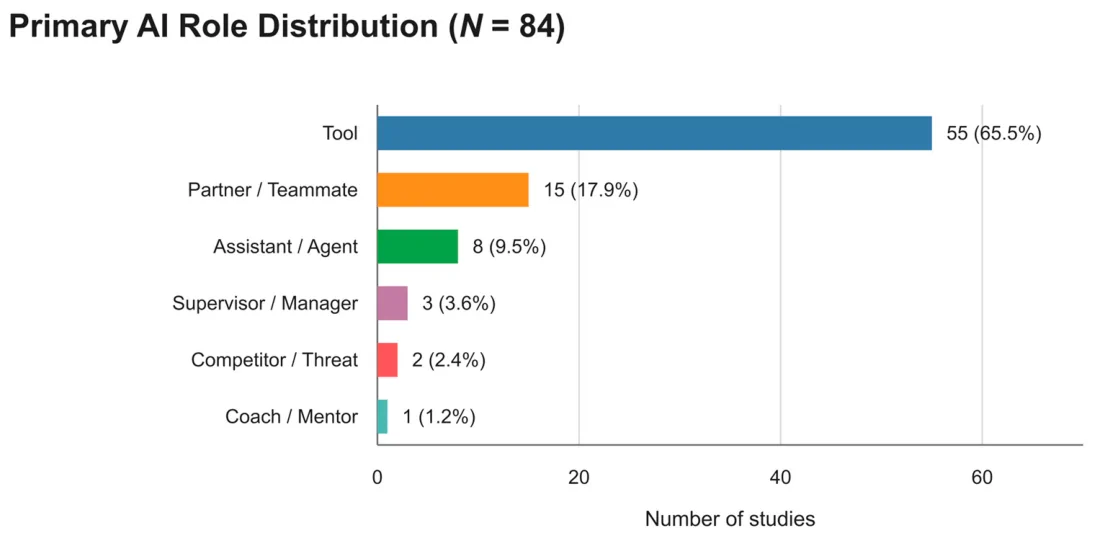
Distribution of CAI roles in the workplace (*N* = 84).

For studies identifying multiple AI roles, the primary role representing the study’s core theoretical contribution or the highest level of anthropomorphic agency was coded to ensure mutually exclusive categories. The role distribution presented in Figure 4 offers a direct answer to RQ1 regarding role evolution. In most studies, CAI remains positioned in a utilitarian capacity. Specifically, 55 studies frame the technology strictly as a tool, and eight as an assistant, agent, or resource. These instrumental conceptualizations are typically theorized through Affordance Theory or the Job Demands–Resources model. This default posture is exemplified by aerospace industry case study of Barba et al. (2025), where large language models served explicitly as content generation engines rather than social counterparts. Consequently, instrumental framing remains heavily dominant in the current literature, with most of the research treating CAI as a utilitarian resource. Nevertheless, a meaningful theoretical turn regarding role elevation has emerged among a minority of studies. Specifically, fifteen studies treat the conversational system as a partner, teammate, or companion, an approach most strikingly visible in Le et al.’s (2025) service sector investigation of human–digital employee collaboration. Furthermore, six studies (7.2%) elevate the technology to the role of supervisor, coach, manager, or mentor, as observed in Terblanche’s (2024) study on how AI coaching is redefining people development. Together, these elevated non-tool roles account for 34.5% of the corpus, representing an emerging and theoretically meaningful direction in the field.

**Figure 5:**
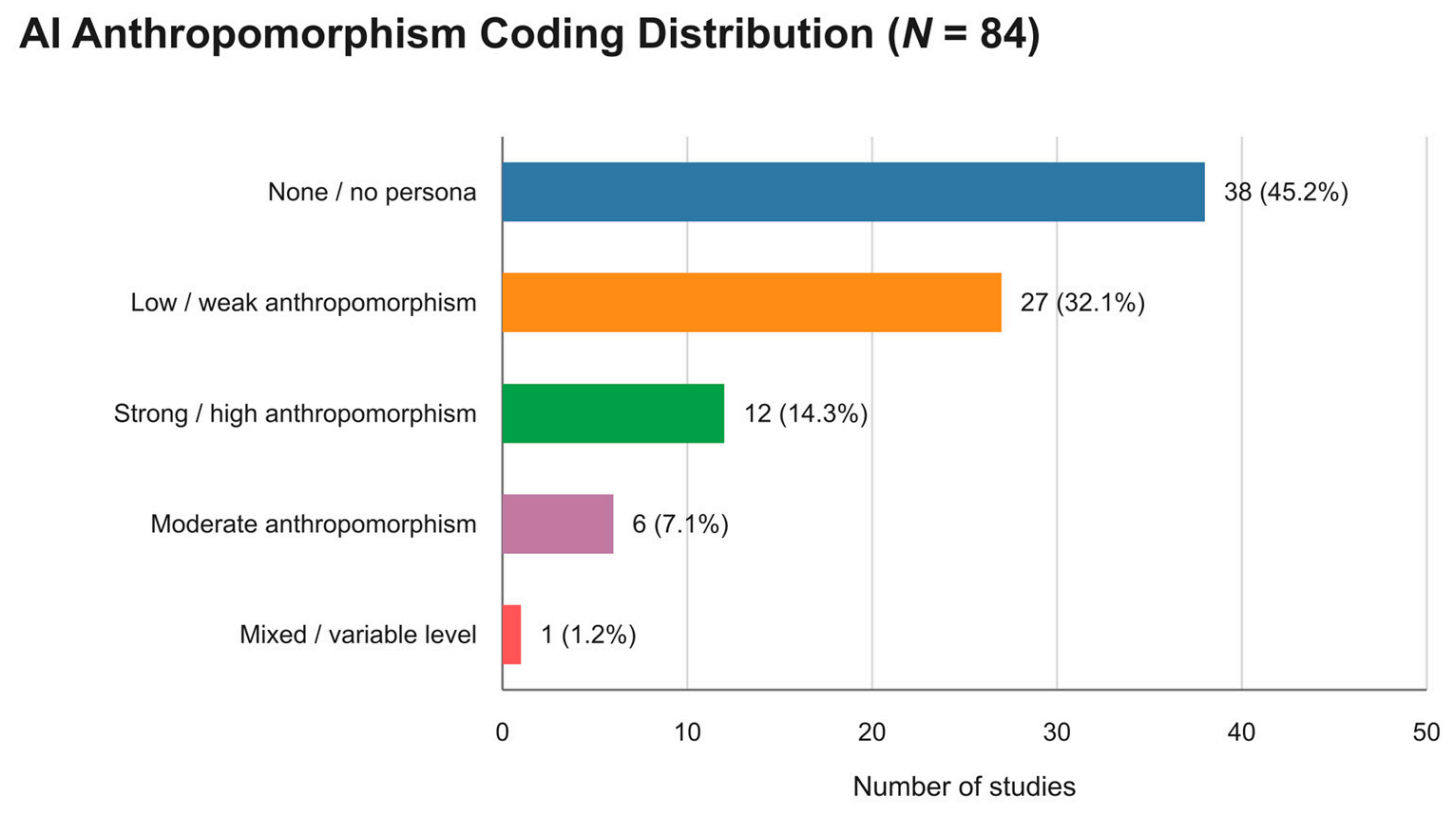
Distribution of CAI anthropomorphism in the workplace (*N* = 84).

Complementing this role shift, the data on anthropomorphism reveals significant variations in design cueing. Within the corpus (see Figure 5), 46 studies (54.8% out of the full corpus of 84 studies) explicitly addressed or manipulated anthropomorphic characteristics. Among this specific subset, 14.3% (*n* = 12 out of the 84 studies) coded the CAI with strong or high anthropomorphic cues. As detailed in the dimensions of anthropomorphism, these highly anthropomorphized systems frequently combined identity cues (e.g., formal names, avatars, and persistent personas) with emotional or empathic expressions, analogous to the paradigm case in Nguyen (2026). Weak or low cues constituted the largest category at 32.1% (*n* = 27 out of 84), typically relying on basic conversational and linguistic fluency (the most prevalent design dimension overall, *n* = 27) without constructing a fully realized persona. Moderate cues accounted for 7.1% (*n* = 6 out of 84). The co-occurrence of role elevation and anthropomorphic cueing provides dense empirical support for the Computers as Social Actors hypothesis. As a CAI system increasingly signals human-like qualities, employees more readily recategorize it from a utilitarian tool to a social counterpart.

## 4. Thematic Findings and Psychological Mechanisms

This section triangulates the empirical findings and theoretical perspectives across the 84 reviewed studies to directly address our three research questions. By integrating the CASA paradigm, STS theory, and JD-R model, we map the psychological mechanisms through which CAI reshapes the employee experience.

### 4.1. From Tool to Social Actor: The Psychological Mechanisms of Anthropomorphism

The quantitative trend from Section 3.2 requires a qualitative explanation. Historically, organizational technology studies defaulted to an instrumentalist reading in which AI functions primarily as a resource to optimize tasks (Barba et al., 2025). Our corpus indicates that this instrumental reading remains dominant, characterizing roughly two-thirds of the field. The remaining third, however, reflects a meaningful psychological recategorization driven by anthropomorphic cues. Consistent with Epley et al.’s (2007) three-factor theory of elicited agent knowledge, effectance motivation, and sociality motivation, conversational AI triggers anthropomorphic processing whenever linguistic fluency, persona continuity, and affective expression co-occur, suggesting that employees implicitly apply social scripts to an artificial system. Pillai et al. (2024), surveying 415 service-sector employees, reported that anthropomorphic chatbot features predicted employee-experience satisfaction through perceived social presence. Nguyen (2026) demonstrated that anthropomorphic features in company-deployed generative AI systems increased knowledge-contributing behaviors and engagement. Furthermore, Gkinko and Elbanna (2022) documented in a longitudinal qualitative study of an enterprise chatbot rollout that employees expressed hope, tolerance, and empathy towards a struggling system. These are emotional stances that can be conceptualized as treating the technology as novice colleagues. Le et al. (2025) extended this observation with service-sector evidence showing that when a digital employee possesses a cohesive persona, coworkers coach its errors and advocate for its inclusion in team rituals, representing a form of anticipatory socialization directed at a machine.

The corpus also highlights non-trivial moderation of this transition. Strong anthropomorphic cues such as name, voice, stable personas, and empathic phrasing are psychologically potent but contextually sensitive. In high-stakes decision settings such as personnel selection, employees simultaneously expect machine-like neutrality and human-like accountability. This creates a double bind that Fisher et al. (2023) identified as a source of distrust among applicants with disabilities. By contrast, weaker cues such as turn-taking and acknowledgement tokens often trigger social interpretation at a lower psychological cost and without generating the same accountability conflict. This dynamic may explain why the largest anthropomorphism category in our corpus is weak rather than strong, representing 36.9% versus 21.4% of the studies respectively. This distribution suggests that anthropomorphic design is not a simple intensity effect but a contextual calibration problem.

Task stakes and decisional authority further shape the role transition and moderate anthropomorphic processing. In contexts where employees retain clear decisional authority and the AI is framed as an assistive counterpart, such as customer service handoffs (Lin et al., 2024) or virtual assistant deployments (Dutta & Mishra, 2025), anthropomorphic cues foster engagement and perceived support. However, when conversational AI is interposed between employees and consequential decisions such as evaluative appraisals, hiring screens, and compensation calculations, the exact same cues can provoke a defensive psychological stance. Song et al. (2025) demonstrated this experimentally by showing that under high-stakes AI appraisals, employees with higher job complexity responded with heightened psychological empowerment, whereas those with lower job complexity responded with increased workplace cheating. This indicates that anthropomorphic framing alone cannot guarantee constructive outcomes when accountability asymmetries loom large. Role elevation therefore depends not only on interface design but also on how responsibility is distributed around the system.

Organizational legitimacy serves as a third recurring moderator in the corpus. Employees engage conversational AI more constructively when its use is openly authorized, supported by training, and embedded in accepted workflow norms. Cho et al. (2025) established this in Korean government agencies, where organizational support mediated the relationship between AI trust and employee adoption. Conversely, where AI use is tacitly permitted but lacks formal governance, employees often rely on the technology privately to meet deadlines while concealing the practice from peers and supervisors. This concealed reliance, often identified as shadow AI use, suppresses knowledge sharing and distorts learning (Chen, 2025). The transition from tool to social actor is therefore partly a design phenomenon and partly a governance phenomenon. Legitimacy operates not merely as an administrative concern but as a psychological precondition for articulating and improving the relationship between employee and machine.

The theoretical significance of this shift is that it reopens the social actor default at an industrial scale. Employees frequently enter the interaction with a social-counterpart hypothesis even while fully aware they are interacting with software. This transition leads to two primary corollaries. First, findings regarding trust, betrayal, and repair in AI interactions must be theorized using interpersonal trust frameworks (McKnight et al., 2002; Rousseau et al., 1998) alongside traditional technology acceptance models. Second, qualitative differences among AI systems, ranging from names and voices to empathic phrasing, function as psychologically decisive design variables rather than mere cosmetic choices, as empirically demonstrated by Pillai et al. (2024). Ultimately, conversational AI becomes a social actor when anthropomorphic cues, task structure, and organizational legitimacy jointly encourage employees to interpret it as a socially relevant counterpart. The underlying mechanism is not technological sophistication alone, but the activation of interpersonal expectations surrounding trust, reciprocity, accountability, and support.

### 4.2. The Three-Dimensional Reshaping of Employee Experience

Once recategorized as a social counterpart, CAI reshapes the employee experience in psychologically distinct yet coupled ways. Drawing on the corpus evidence and extending prior schemas such as Filippelli et al.’s (2026), we distinguish three interdependent dimensions of the employee experience: cognitive, emotional–social, and career–ethical. Table 3 synthesizes the double-edged effects across these dimensions, while the subsequent analysis explains how they operate and reinforce one another.

At the cognitive level, conversational AI frequently functions as an augmentation resource that facilitates a human intelligence augmentation effect. Lin et al. (2024) demonstrated in a mixed methods study that chatbot support increased customer service agents’ diagnostic accuracy and task efficiency. Similarly, Brachten et al. (2020) reported measurable reductions in cognitive load during routine inquiry handling. Freed from routine processing, employees can redirect cognitive resources toward higher-order activity such as problem-finding, as Sabbah and Li (2025) documented in human–LLM collaboration scenarios. However, this reconfiguration carries psychological cost. Chen (2025) found that skill threat perception, defined as the anticipation that accumulated domain expertise will be devalued, significantly suppressed knowledge-sharing behavior even when AI self-efficacy remained high. Furthermore, Sarkar (2026) argues that the cognitive locus shifts from executing tasks to crafting prompts and verifying probabilistic outputs, crafting a new layer of cognitive burden and decision fatigue. The cognitive experience is therefore characterized by simultaneous augmentation and vigilance.

The emotional and social evidence reveals a similar duality. CAI furnishes a form of interactional warmth unusual in organizational life by being continuously available, non-judgmental, and infinitely patient. Qi et al. (2025) found that generative AI use was positively associated with digital performance partly through reduced emotional exhaustion. Gkinko and Elbanna (2022) even documented employees extending hope, tolerance, and empathy to a struggling enterprise chatbot. While these findings validate the technology as a psychological resource, reliance on artificial systems can fray the organizational social fabric. Baygi and Huysman (2026) warned that when employees learn to consult the system in preference to colleagues, the informal lattice of help-seeking and reciprocity weakens. Li et al. (2025) corroborated this with survey evidence showing that employee collaboration with generative AI was associated with a decline in self-reported frequencies of actual helping behavior among colleagues. Based on the laziness attribution and responsibility-avoidance attribution, Wu and Jiao (2026) similarly found that higher perceived employee–AI collaboration correlated with lower self-reported coworker helping behavior. While these findings rely on self-reported behavioral measures rather than direct objective tracking, they suggest a potential risk to the organizational social fabric. The critical factor governing these outcomes is whether the technology is positioned as an augmenting presence that enriches peer exchange or a substituting presence that replaces human relationships.

The career and ethical aspects demonstrate significant changes in meaningful work, fairness perception, and behavioral adaptation. Callari and Puppione (2025) identified configurations where employees successfully reconstructed meaningful work around AI-assisted practices. Dutta and Mishra (2025) showed that supportive AI virtual assistants increased perceived procedural fairness and work engagement. Singh et al. (2025) similarly demonstrated that conversational tools could enhance leaders’ ability to translate inclusion strategies into daily behaviors. Nevertheless, the opacity and asymmetry of these systems produce emergent ethical risks. Fisher et al. (2023) highlighted algorithmic exclusion, arguing that AI-enabled screening systems can systematically disadvantage applicants with disabilities whose communication profiles deviate from training data norms. Hai et al. (2025) found that high-intensity digital demands created by generative AI produced work alienation, which subsequently increased employee expediency. Saluja et al. (2025) identified generative AI loafing, a phenomenon where employees transfer cognitive effort wholesale to the machine, leading to the atrophy of critical thinking. Additionally, Song et al. (2025) experimentally demonstrated that high-stakes AI appraisal contexts can raise employees’ propensity to engage in workplace cheating. Crucially, these negative outcomes represent structural adaptations rather than mere technical failures, highlighting that artificial intelligence systems are not neutral. Many ethical risks arise directly from design choices, training data biases, opacity, automated evaluation, and asymmetric accountability, rather than simply poor employee use. It is important to distinguish the strength of evidence across these negative outcomes. While phenomena such as workplace cheating, work alienation and employee expediency are currently supported by robust quantitative empirical evidence, the concepts of organizational social fabric erosion and generative artificial intelligence loafing rely primarily on emerging conceptual frameworks or qualitative case observations. Consequently, these latter risks possess a more tentative empirical foundation and require further rigorous validation to establish their generalizability across the field.

These three dimensions do not operate independently. A key synthetic observation across the corpus is that they are psychologically coupled. Rather than implying a verified sequential causal chain, the literature points to a proposed conceptual pathway where a cognitive skill threat appraisal may align with emotional career anxiety, which can in turn relate to a lowered threshold for ethical expediency. Read together, studies by Chen (2025), Hai et al. (2025), and Saluja et al. (2025) trace a recurring pattern where the perceived devaluation of expertise is positively associated with increased anxiety, weakened identification with work and lowered resistance to loafing or expedient behaviors. This coupled experience structure implies that single-dimensional interventions, such as isolated AI literacy training, are insufficient. Instead, organizations require integrated governance strategies that address the cognitive, emotional, and ethical strands simultaneously.

### 4.3. Antecedents, Pathways, and the Integrated Model

#### 4.3.1. The Three-Layered Antecedent Structure

Entry into deep human–AI collaboration is conditioned by a three-layered antecedent structure. At the individual layer, AI literacy, AI self-efficacy, and identity security emerge as central drivers that influence whether employees treat conversational AI as an aid or a threat. As supporting insights from the broader AI collaboration literature, Xu et al. (2025) revealed that AI collaboration strengthened employees’ AI identity, enabling them to recognize their identity as AI collaborators and focus on in-role tasks, thereby reducing cyberloafing. Chen (2025) stated that AI self-efficacy buffered the negative effect of skill threat on knowledge sharing, while Saluja et al. (2025) suggested that limited critical engagement can intensify over-reliance. At the organizational layer, digital trust climate, training, and perceived organizational support govern whether the use of the technology is experienced as legitimate and developmental or as imposed and risky. Cho et al. (2025) demonstrated that organizational support mediated the link between trust in AI and chatbot adoption. Conversely, Baygi and Huysman (2026) document that laissez-faire organizational stances can precipitate both system misuse and inter-employee trust erosion. At the task layer, the complexity of work and the degree of discretion retained by employees shape the role the system is permitted to take. Large-scale recruitment screening, document generation, IT self-service, and creative composition each produce a distinct collaboration mode. These three layers jointly determine employees’ initial construal of the technology and shape the downstream psychological pathway.

A further antecedent that requires explicit theoretical attention is the employee’s professional identity prior to the technology encounter. Emerging evidence within the corpus suggests that in knowledge-intensive professions, employees with strong, stable professional identities tend to engage the system as a tool or collaborator within a well-defined repertoire. In contrast, those with weaker or transitional identities can appear more vulnerable to the identity threat of skill devaluation (Chen, 2025). This pattern suggests that professional identity stability appears to function as a psychological resource, potentially buffering the demand-like features of the technological encounter, and enables employees to process novelty without destabilization.

Importantly, these antecedents operate as configurations rather than isolated predictors. High AI literacy in a low-trust climate often produces concealed or cynical use, where employees utilize the system but protect their core work from it (Chen, 2025). High trust without sufficient literacy can produce over-reliance that eventually erodes skill. The most constructive forms of collaboration emerge when employee capability, supportive climate, and task fit develop simultaneously (Callari & Puppione, 2025). This configurational dynamic helps explain why outcomes vary so sharply across different organizational settings.

#### 4.3.2. Positive and Negative Outcomes

The outcomes of these interactions are distinctly double-edged. Positive outcomes include measurable performance gains (Qi et al., 2025), enhanced incremental and radical creativity (Zhang et al., 2025), psychological empowerment (Song et al., 2025), and strong procedural fairness perceptions (Dutta & Mishra, 2025; Singh et al., 2025). Negative outcomes cluster along three recurring pathways. The first is a strain pathway, where intensified digital demands yield alienation and emotional exhaustion (Hai et al., 2025). The second is a moral pathway, where alienation combines with hindrance stressors to produce GenAI loafing (Saluja et al., 2025) and workplace cheating (Song et al., 2025). The third is a relational pathway, where the system substitutes for peer help-seeking, thereby eroding spontaneous helping and knowledge-sharing (Baygi & Huysman, 2026; Li et al., 2025). Technical limitations, such as hallucination or knowledge-based gaps, function primarily as boundary conditions that shape how these pathways unfold rather than directly constituting the psychological outcomes themselves.

The temporal unfolding of these outcomes represents a critical emerging theme. Gkinko and Elbanna’s study (2022) captured an early phase characterized by hope and tolerance, essentially a psychological honeymoon where employees extended generous benefit of the doubt to a struggling system. Later phases revealed a shift toward calibrated expectation and occasional disillusionment. This trajectory echoes the established literature on interpersonal-trust development (Rousseau et al., 1998) and demonstrates that the relationship between employees and artificial systems evolves through identifiable stages.

From a psychological mechanism standpoint, the negative pathways are unified by a common feature: they represent responses to structural asymmetries. CAI introduces or intensifies the asymmetry of information between humans and algorithms, the asymmetry of pace between machine output and human verification, and the asymmetry of accountability between opaque systems and transparent employees. When these asymmetries are balanced by explanation, employee discretion, and shared norms, augmentation is highly likely. When they remain unbalanced, harm typically follows.

#### 4.3.3. Integrated Conceptual Model

**Figure 6:**
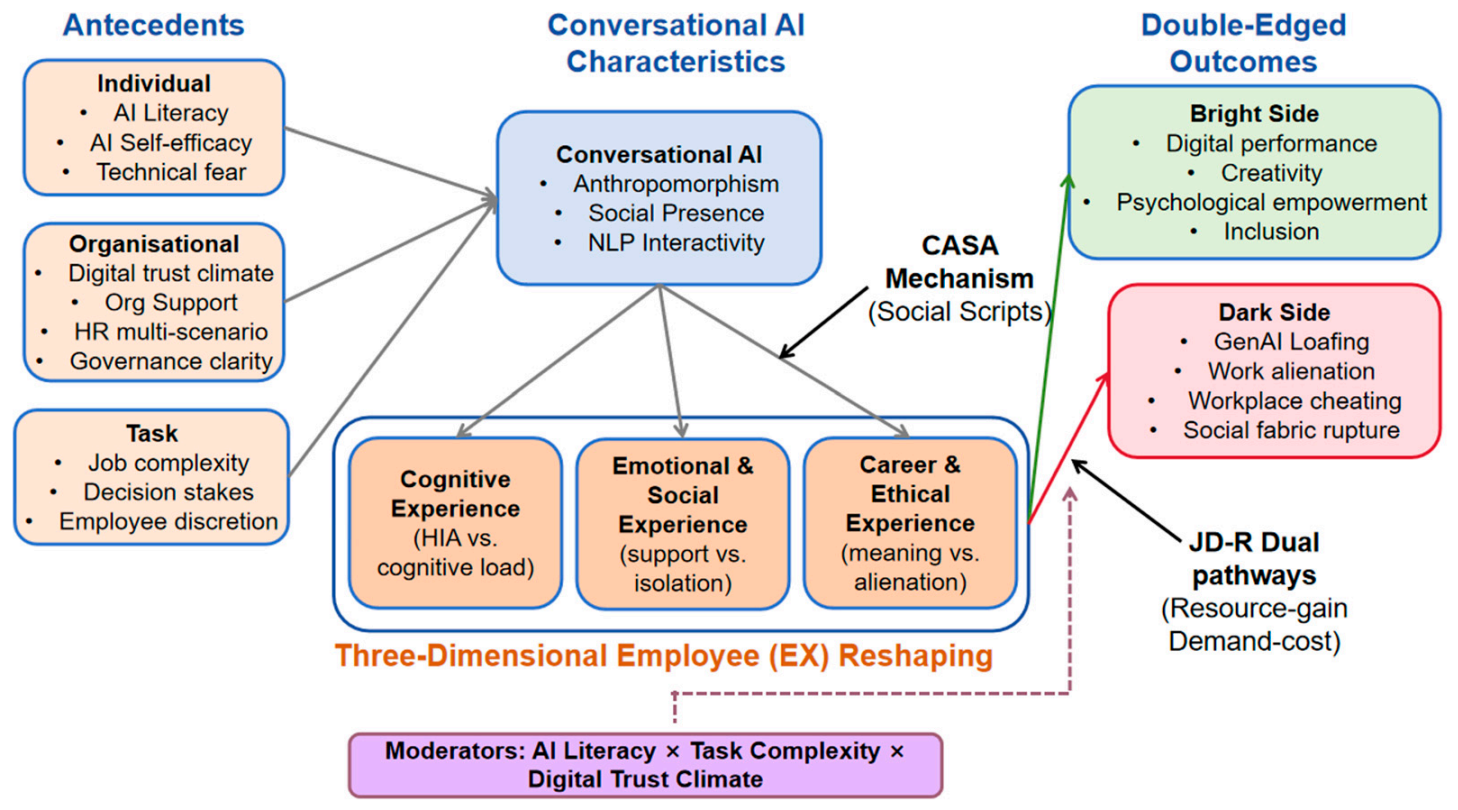
Integrated conceptual model of employee experience reshaping via conversational artificial intelligence. This model synthesizes the corpus findings through three theoretical lenses. First, individual, organizational, and task antecedents drive the interaction. Second, via the Computers as Social Actors (CASA) mechanism, anthropomorphic cues may activate a three-dimensional (cognitive, emotional, and ethical) psychological reshaping. Third, following the Job Demands–Resources (JD-R) dual pathways, this reshaped experience is theorized to link to either enabling bright-side outcomes or detrimental dark-side adaptations, moderated by contextual trust and task complexity.

Figure 6 synthesizes the corpus evidence into an integrated conceptual model. The model identifies a logical progression where three-layered antecedents (individual, organizational, and task) feed into the system’s role profile and anthropomorphic features. Through the activation of social scripts, the technology then engages the cognitive, emotional–social, and career–ethical dimensions of the employee experience. Operating through the dual pathways of resource gain and demand cost, this process generates either enabling or dark-side outcomes. The model is deliberately psychologically anchored; its focal point is the employee’s internal psychological experience rather than the firm’s operational efficiency. Because most primary studies in this corpus employ cross-sectional designs the pathways illustrated in Figure 6 represent theoretical propositions and conceptual interpretations derived from the synthesis rather than established causal mechanisms. Future longitudinal research is strictly required to validate these sequences.

#### 4.3.4. Research-Question Cross-Mapping

Table 4 maps each research question to its core theoretical lens, the corresponding result section, and the key evidence pattern, making the analytical logic of the synthesis fully transparent.

## 5. Discussion

### 5.1. Dialog with Prior Syntheses

The synthesized findings engage substantively with anchor reviews in the field, indicating a nuanced conceptual shift. For example, Vrontis et al. (2022) and Kambur and Yildirim (2023) mapped artificial intelligence primarily as an enabling infrastructure for human resource management processes, often emphasizing intelligent efficiency. While this instrumental perspective remains the dominant paradigm across roughly two-thirds of the reviewed corpus, the post-ChatGPT literature reveals an emerging yet theoretically meaningful shift where conversational AI is increasingly conceptualized as a social actor rather than mere infrastructure.

Consequently, efficiency gains are sometimes accompanied by potential risks to the organizational social fabric (Baygi & Huysman, 2026). This pattern necessitates an internalized social-cost audit within the intelligent management discourse. Furthermore, Bankins et al. (2024) called for employee-level psychological research, which this synthesis operationalizes by providing a three-dimensional coordinate system that captures cross-dimensional coupling. While Budhwar et al. (2023) and Dwivedi et al. (2023) forecasted opportunities and dangers in commentary pieces, this empirical synthesis specifies these tentative mechanisms, documenting phenomena such as work alienation and generative AI loafing. Finally, while Filippelli et al.’s (2026) three-dimensional well-being schema informed the early stage of this research, the current corpus support elevates career–ethical concerns to a peer dimension rather than a residual category.

Taken together, these dialogs suggest that the field is undergoing a theoretical reorientation. The relevant question is no longer whether employees adopt the technology, but how they interpret, negotiate, and morally accommodate a conversational system that increasingly resembles a workplace counterpart. A purely technology acceptance framing is now inadequate. It must be supplemented by theories of social categorization, interpersonal trust, moral disengagement, and resource conservation. Crucially, while previous anchor reviews were written from distinct disciplinary bases such as information systems or innovation management, the evidence suggests that no single discipline can fully encompass this phenomenon. Psychology occupies a privileged analytical position because the core mechanisms, such as construal, attribution, trust, and moral disengagement, are fundamentally psychological rather than strictly organizational or technological.

### 5.2. Theoretical Contributions

This synthesis contributes to the psychology of technology at work in four ways. First, it complements the technology adoption literature by integrating psychological mechanisms. The review demonstrates that the workplace impact of conversational systems is mediated by how employees construe the system, a construal systematically predicted by anthropomorphic design cues (Nguyen, 2026; Pillai et al., 2024). This positions the Computers as Social Actors paradigm as an essential import into organization behavior theorizing. Second, it develops an integrated, three-dimensional coordinate system for the employee experience, detailing synthesized cross-dimensional associations and thereby advancing Bankins et al.’s (2024) conceptual agenda into testable propositions. Third, it consolidates dispersed evidence on dark-side outcomes such as work alienation (Hai et al., 2025), GenAI loafing (Saluja et al., 2025), workplace cheating (Song et al., 2025), and social fabric rupture (Baygi & Huysman, 2026), delineating these employee-side behavioral adaptations from technology-end boundary conditions like system hallucinations. Fourth, by fixing the unit of analysis strictly at the direct human party of the human–AI interaction, we establish a methodological discipline that prevents disparate outcomes from being conflated.

### 5.3. Practical Implications

Practical translation of these findings should proceed with psychological design logic rather than efficiency logic alone. Organizational efforts to implement CAI framed solely around productivity targets tend to accelerate dark-side pathways because they intensify structural asymmetries without creating psychological slack for sense-making. Three practice principles follow from this review. First, organizations should design for system-plus-peer collaborative architectures rather than individualistic ones, ensuring that task engagement preserves opportunities for spontaneous collegial interaction and prevents the displacement of peer exchange (Baygi & Huysman, 2026). Second, systems must be deployed strategically to counter algorithmic exclusion. This requires auditing high-stakes evaluative tools for disparate impacts on diverse workforce populations, including applicants with disabilities, older workers, non-native speakers, and employees subject to algorithmic monitoring, ensuring technology enhances rather than merely preserves inclusion (Singh et al., 2025). Third, organizations should institute structured social-cost audits alongside standard performance metrics. Operationalizing these audits requires utilizing a mixed methods approach: organizational social network analysis (SNA) can map shifting consultation patterns and help-seeking frequencies; anonymous surveys can track knowledge-sharing behaviors and perceived psychological safety; and HR analytics combined with qualitative focus groups can uncover changes in broader human-to-human collaboration. Furthermore, organizations must recognize that artificial intelligence literacy training alone is insufficient. Many emerging risks are structurally linked to system design, task redesign, surveillance practices, and accountability arrangements, necessitating comprehensive governance frameworks. Employees respond more constructively when responsibility for verification is clear and when using conversational tools is explicitly authorized rather than covertly practiced.

### 5.4. Limitations

While a comprehensive map of the current literature is proposed, the interpretive scope of this synthesis is bounded by several intrinsic constraints. First, the literature search was explicitly restricted to the Web of Science Core Collection and English-language publications. Because conversational AI is an inherently interdisciplinary phenomenon encompassing human–computer interaction, computer science, and information systems, this bounded search strategy likely excluded relevant insights published in prominent technical conference proceedings (e.g., ACM or IEEE) or specialized psychological databases (e.g., PsycINFO). Future syntheses should expand beyond the SSCI index to capture a more comprehensive, multidisciplinary reality. Second, the coding protocol prioritized the primary construct or the highest level of anthropomorphic agency in studies featuring multiple AI roles. While theoretically justified to capture the maximum social cueing of CAI and prevent duplicative coding, this decision may have inherently skewed the synthesized distribution toward more socially agentic interpretations of conversational AI, potentially underrepresenting more passive or purely tool-like roles in multi-role research. Third, the review protocol was not pre-registered, an increasingly important norm in psychological research. Fourth, quality appraisal inevitably involved subjective elements, particularly when evaluating conceptual papers. Finally, most primary studies in the corpus rely on cross-sectional designs, which constrains inferences regarding the temporal dynamics of trust, anthropomorphic attachment, and alienation. Sixth, the reliance on very recent (2025–2026) and early-access literature means that emerging constructs such as GenAI loafing, social fabric rupture, and workplace cheating lack widespread empirical replication, rendering these findings preliminary rather than definitive.

## 6. Conclusions and Future Directions

Amid a wave of conversational artificial intelligence rapidly reorganizing everyday work, the deep question for the psychology of work is not whether artificial systems will replace employees but what kind of colleagues humans are becoming to the machines and, through them, to one another. The synthesis of 84 reviewed studies demonstrates that through anthropomorphic cues and advanced interactivity, these systems have already begun to renegotiate the social boundaries of the workplace. Three critical integrative insights emerge. First, the synthesis points to a possible pathway where cognitive, emotional–social, and career–ethical dimensions are closely aligned; within this framework, perceived skill threats may be associated with elevated anxiety, which can in turn relate to a lower threshold for ethical expediency (Chen, 2025; Hai et al., 2025; Saluja et al., 2025). Second, efficiency-first deployments risk silently depleting the informal mutual-help networks upon which organizational resilience relies (Baygi & Huysman, 2026; Li et al., 2025). Third, emergent dark-side phenomena such as generative artificial intelligence loafing and workplace cheating are conceptualized as behavioral adaptations to asymmetric human–AI arrangements and high digital demands (Fisher et al., 2023; Saluja et al., 2025; Song et al., 2025).

These insights rest on a rapidly evolving evidence base that demands vigorous future research across several frontiers. The field urgently requires longitudinal designs using diary, experience sampling, and social network methods to trace how human–AI trust is formed, violated, and repaired across the collaboration lifecycle. Additionally, research must investigate work–family spillover to determine whether the reduction in workplace emotional labor translates into family enrichment or generates persistent digital anxiety. Algorithmic fairness and cross-cultural comparisons represent further critical frontiers. Researchers must specifically study the experiences of older workers, applicants with disabilities, and non-mainstream language users, while also examining how differing institutional and collectivistic–individualistic contexts shape psychological pathways beyond the predominantly Western and Chinese samples currently dominating the literature.

A cross-cutting frontier concerns measurement and individual differences. The construct of anthropomorphism, alongside conversational artificial intelligence-specific trust and digital work expediency, is currently operationalized with significant heterogeneity. The psychological validity of cross-study comparisons depends entirely on the development of shared, robust psychometric instruments adapted for occupational settings. Furthermore, future inquiry must move beyond average effects to consider how individual traits, such as an inherent tendency toward anthropomorphism or the need for decisional autonomy, moderate employee responses to artificial systems.

The wave of conversational artificial intelligence will not slow. How it reshapes the psychology of work depends on whether the academic community can study it with the theoretical discipline and social imagination the moment demands. The central empirical lesson of this synthesis is that these systems appear to generate complex dual pathways of flourishing and strain across diverse workplace settings. The conceptual lesson is that these outcomes are patterned by whether organizations and researchers recognize conversational artificial intelligence as a new class of social counterpart and accept the psychological responsibilities such recognition entails. By shifting the focus from narrow productivity gains to internal experience reshaping, this study delivers three primary contributions: a bounded systematic synthesis of the literature, an integrated three-dimensional framework of employee experience, and a structured set of cautious propositions designed to guide future longitudinal and experimental research.

## Figures and Tables

**Table 1:** Comparison of published SLRs and the present review.

Author & Year	Research Focus	Primary Level of Analysis	Theoretical Lens/Organizing Logic	Primary Focus and Boundary
Vrontis et al. (2022)	Intelligent automation strategies and organizational performance.	Macro (Organizational) & Workforce	Multidisciplinary HRM-IM-IB-GM synthesis.	Treats technology as infrastructure focusing on organizational performance rather than micro-level psychological role transitions.
Jatobá et al. (2023)	AI adoption in HRM functions (recruitment, training, etc.).	Meso (Strategic/Managerial)	Cluster-based content analysis of adoption paths.	Emphasizes managerial adoption pathways bounding the focus away from the fine-grained lived experience of end-users.
Úbeda-García et al. (2025)	Thematic mapping of AI–HRM interactions (automation to personalization).	Macro & Meso (Field/Organizational)	Bibliometric science mapping.	Maps broad thematic fields without operationalizing a specific micro-level psychological framework.
Kekez et al. (2025)	Bias and discrimination in AI-enabled HRM decisions.	Algorithmic & Policy	Fairness and ethical implications.	Isolates the ethical dimension specifically bounding the review to fairness rather than multi-dimensional employee experience.
Alherimi et al. (2025)	AI advancements in Green HRM and sustainability.	Macro (Sectoral/Organizational)	Sustainability and adoption models.	Bounds focus to sustainability routines rather than the general psychological transition of conversational agents into social actors.
The Present Review	Psychological mechanisms reshaping employee experience via CAI.	Micro (Interpersonal/Psychological)	CASA + STS + JD-R framework.	Moves from “AI-as-infrastructure” to “AI-as-social-actor”, providing an integrated antecedent-process-outcome model.

**Table 2:** Theory-driven 16-dimension coding framework.

Level-1 Category	Level-2 Dimension	Theoretical Anchor & Coding Description
Basic Study Characteristics	1. Publication year	Year of publication
	2. Journal & discipline	Specific journal of publication
	3. Document type	Empirical/Review/Conceptual
Theoretical & Methodological Foundation	4. Dominant theory	The primary theoretical lens
	5. Research method	Quantitative/Qualitative/Mixed/Conceptual
	6. Sample & context	Sample size and organizational setting
Role & Attributes of Conversational AI	7. Technology form	Generative AI/LLMs (ChatGPT)/Enterprise Chatbot
	8. Anthropomorphism	Strong/Moderate/Weak/None
	9. AI role	Tool/Partner/Supervisor/Assistant/Competitor
Core Dimensions of Employee Experience (EX)	10. Cognitive EX	Cognitive Relief/Cognitive Augmentation/Cognitive Depletion/Cognitive Strain
	11. Emotional–social EX	Positive (Trust/Empathy)/Negative (Anxiety/Fear)/Social Alienation/Psychological Safety
	12. Career–ethical EX	Fairness/Justice/Privacy & Data Security/Job Insecurity/Displacement/Professional Identity
Antecedents & Outcomes of Human–AI Interaction	13. Individual antecedents	AI Literacy/Digital Skills/Trust Propensity/Personality (Openness)/Prior Expectations
	14. Organizational & task antecedents	Task Complexity/Org Support/Climate/Governance & Policy/Role/Workflow Design
	15. Positive outcomes	Productivity & Efficiency/Decision Quality/Well-being & Engagement/Creativity/Enhanced Opportunities
	16. Negative outcomes	Deskilling & Over-reliance/Resistance/Abandonment/Job Displacement Anxiety/Bias & Inequality

Note: This table presents a distilled summary of the operational criteria. For the complete coding dictionary, detailed boundary protocols, and representative examples, please refer to Supplementary Material S1.

**Table 3:** The double-edged effects of conversational AI on employee experience.

Dimension	Bright Side	Dark Side	Primary Evidence Base	Representative Studies
Cognitive	Human intelligence augmentation; reduced cognitive load; problem-finding	Skill threat; prompt-engineering burden; judgment dependence	Mixed methods: Qualitative + quantitative survey	Lin et al. (2024)
			Quantitative experiment	Brachten et al. (2020)
			Conceptual	Sabbah and Li (2025)
			Quantitative survey	Chen (2025)
			Conceptual	Sarkar (2026)
Emotional–Social	24/7 emotional support; burnout relief; tolerance & empathy	Social fabric rupture; declining help-seeking	Quantitative PLS-SEM + fsQCA	Qi et al. (2025);
			Qualitative case study	Gkinko and Elbanna (2022)
			Conceptual	Baygi and Huysman (2026)
			Quantitative survey	Li et al. (2025)
Career–Ethical	Meaningful work; bias reduction; inclusion	Algorithmic exclusion; work alienation; GenAI loafing; workplace cheating	Quantitative	Callari and Puppione (2025)
			Quantitative field study	Dutta and Mishra (2025)
			Mixed methods: Qualitative and quantitative	Singh et al. (2025)
			Conceptual	Fisher et al. (2023)
			Quantitative	Hai et al. (2025)
			Conceptual	Saluja et al. (2025)
			Quantitative survey	Song et al. (2025)

**Table 4:** Cross-mapping of research questions, theoretical lenses, and result sections.

RQ	Theoretical Lens	Result Section	Key Evidence
RQ1 Role evolution	CASA and Anthropomorphism	Section 3.2 and Section 4.1	CAI increasingly shifts from tool to partner, evaluator, or social actor under anthropomorphic cueing and supportive governance.
RQ2 Experience reshaping	STS and JD-R	Section 4.2 and Table 3	The technology reshapes employee experience across cognitive, emotional–social, and career–ethical dimensions, demonstrating coupled bright-side and dark-side effects.
RQ3 Antecedents and outcomes	JD-R and STS	Section 4.3 and Figure 6	Individual, organizational, and task antecedents channel employees toward distinct pathways of augmentation, strain, moral adaptation, or social fabric erosion.

## Data Availability

The original contributions presented in this study are included as Supplementary Materials. Further enquiries can be directed to the corresponding author.

## Supplementary Materials

The following supporting information can be downloaded at: https://www.mdpi.com/article/10.3390/bs16091704/s1, Supplementary Material S1: Detailed coding dictionary and operationalization of variables; Supplementary Material S2: Comprehensive evidence map of the included studies.
